# Causes of delay in door-to-balloon time in south-east Asian patients undergoing primary percutaneous coronary intervention

**DOI:** 10.1371/journal.pone.0185186

**Published:** 2017-09-21

**Authors:** Wen Jun Sim, An Shing Ang, Mae Chyi Tan, Wen Wei Xiang, David Foo, Kwok Kong Loh, Fahim Haider Jafary, Timothy James Watson, Paul Jau Lueng Ong, Hee Hwa Ho

**Affiliations:** 1 Department of Cardiology, Tan Tock Seng Hospital, Singapore, Singapore; 2 Clinical Research & Innovation Office, Tan Tock Seng Hospital, Singapore, Singapore; University of Tampere, FINLAND

## Abstract

**Objective:**

To evaluate causes and impact of delay in the door-to-balloon (D2B) time for patients undergoing primary percutaneous coronary intervention (PPCI).

**Subjects and methods:**

From January 2009 to December 2012, 1268 patients (86% male, mean age of 58 ± 12 years) presented to our hospital for ST-elevation myocardial infarction (STEMI) and underwent PPCI. They were divided into two groups: Non-delay defined as D2B time **≤** 90 mins and delay group defined as D2B time > 90 mins. Data were collected retrospectively on baseline clinical characteristics, mode of presentation, angiographic findings, therapeutic modality and inhospital outcome.

**Results:**

202 patients had delay in D2B time. There were more female patients in the delay group. They were older and tend to self-present to hospital. They were less likely to be smokers and have a higher prevalence of prior MI. The incidence of posterior MI was higher in the delay group. They also had a higher incidence of triple vessel disease.

The 3 most common reasons for D2B delay was delay in the emergency department (39%), atypical clinical presentation (37.6%) and unstable medical condition requiring stabilisation/computed tomographic imaging (26.7%). The inhospital mortality was numerically higher in the delay group (7.4% versus 4.8%, p = 0.12).

**Conclusions:**

Delay in D2B occurred in 16% of our patients undergoing PPCI. Several key factors for delay were identified and warrant further intervention.

## Introduction

Primary percutaneous coronary intervention (PPCI) [1] has emerged as the preferred therapy for ST-elevation myocardial infarction (STEMI) if performed in a timely fashion and it is the first-line reperfusion therapy in Singapore. The promptness of PPCI can be measured by using the door-to-balloon (D2B) time. It is also an indicator of quality of care of PPCI program and is predictive of outcome. A D2B time > 90 minutes has been shown by several studies [2–5] to be associated with higher mortality. Based on these data, the American College of Cardiology and American Heart Association (ACC/AHA) STEMI guidelines [6] recommends D2B ≤ 90 minutes for PPCI for STEMI. Several studies [7–11] have described the reasons for D2B time delay and highlighted patient demographic characteristics and certain issues unique to their patient population. Prior studies were mostly conducted in the Western population but the current situation in Singapore is unknown. We evaluated the D2B time in STEMI patients presenting to our hospital, elucidate the reasons for delay and identify opportunities to improve quality of care.

## Material and methods

This is a retrospective study of 1278 patients who underwent PPCI in our hospital from January 2009 to December 2012. We included patients who had electrocardiographic (ECG) evidence of STEMI, presented within 12 hours of symptom onset to our emergency department (ED). Excluded were 10 patients with absence of ST elevation on ECG and those with inadequate documentation of arrival or reperfusion times. Hence, 1268 patients were included in the final analysis.

Our invasive cardiac catheterisation laboratory (> 1300 PCI/year) provides 24 hour PPCI services for STEMI patients with a PPCI volume of > 350 cases/year. A STEMI protocol [12] has been established in our institution in which the emergency medicine physician directly activate the catheterization laboratory, a single call to a central operator to activate the on-call team and staff are expected to arrive within 20 minutes after being activated. Data were retrieved on the baseline clinical characteristics, ECG, D2B time, angiographic findings, mode of treatment and clinical outcome. D2B time was defined according to ACC definition as the interval between the hospital arrival time and the time of restoration of Thrombolysis in Myocardial Infarction (TIMI) 3 flow by whatever device used. Delay was defined by cut-off of D2B time > 90 minutes. We compared the clinical characteristics of patients in the delay and non-delay group to identify patient-related factors leading to D2B delay and also evaluated its impact on inhospital mortality. Inhospital mortality is defined as death from any cause during index hospitalization. We also determine the causes of D2B delay of each affected patient by reviewing the relevant medical records.

Continuous variables were expressed as mean ± standard deviation and tested with Student's *t-*test. Categorical variables were expressed as counts and percentages with chi square test or Fisher’s exact test. Calculations were performed using SPSS software (version 16.0; SPSS,Inc., Chicago, Illinois). All *p-*values were 2-sided and *p*-values < 0.05 were considered statistically significant. The study was reviewed and approved by the National Healthcare Group (NHG) Institutional Review Board, Singapore.

## Results and discussion

Table 1 summarizes the baseline clinical characteristics, D2B time, angiographic findings and clinical outcomes.

**Table 1:** Baseline clinical characteristics, D2B time, angiographic findings and clinical outcomes of the study population.

	Overall	Delay	Non-Delay	p
	(N = 1268)	(N = 202; 16%)	(N = 1066; 84%)	
Age, years	58.4 ± 12.2	60.5 ± 13.2	58 ± 12	0.008*
Male:Female,n,%	1096: 172	162:40	934: 132	0.007*
	(86: 14)	(80: 20)	(88: 12)	
**Ethnicity**,n,%:				
Malay	184 (14.5)	22 (10.9)	162 (15.2)	0.12
Chinese	819 (64.6)	131 (64.8)	688 (64.5)	1.00
Indian	202 (15.9)	39 (19.3)	163 (15.3)	0.17
Others	63 (5)	10 (5.0)	53 (5.0)	1.00
Mean symptom onset to reperfusion, mins	251.3 ± 218	322.4 ± 221	240.3 ± 215	< 0.0001*
Mean D2B, mins	68 ± 35	126 ± 46	57 ± 16	< 0.0001*
Median D2B, mins	60	112	56	
Smoker,n,%	671 (53)	90 (45)	581 (54.5)	0.01*
Diabetes mellitus,n,%	365 (28.8)	68 (34)	297 (28)	0.11
Hypertension,n,%	664 (52.4)	111 (55)	553 (52)	0.44
Hyperlipidemia,n,%	680 (53.6)	114 (56)	566 (53)	0.4
Prior MI,n,%	128 (10.1)	30 (15)	98 (9.2)	0.02*
Prior PCI,n,%	103 (8.1)	17 (8.4)	86 (8.1)	0.9
Prior CABG,n,%	6 (0.5)	2 (1.0)	4 (0.4)	0.24
Self-present: EMS,n,%	495: 772	104: 98	391: 674	0.0001*
	(39: 61)	(51.5: 48.5)	(36.7: 63.2)	
Office hours: After office hours,n,%	477: 790	62: 140	415: 650	0.03*
	(38: 62)	(31: 69)	(39: 61)	
**Type of AMI**,n,%				
Anterior	595 (46.9)	99 (49)	496 (46.5)	0.53
Inferior	599 (47.2)	76 (37.6)	523 (49.1)	0.003*
Posterior	19 (1.5)	8 (4.0)	11 (1.0)	0.006*
**No.of VD**,n,%				
1	412 (32.5)	64 (31.7)	348 (33)	0.74
2	415 (32.7)	55 (27.2)	360 (34)	0.06
3	430 (33.9)	83 (41.0)	347 (33)	0.03*
**Target vessel**,n,%				
LAD	604 (47.6)	97 (48)	507 (47.6)	0.93
RCA	502 (39.6)	69 (34)	433 (40.6)	0.09
LCX	115 (9.1)	24 (12)	91 (8.5)	0.14
Left main	37 (2.9)	10 (5)	27 (2.5)	0.06
Cardiogenic Shock,n,%	258 (20.3)	37 (18.3)	221 (20.7)	0.5
Inhospital mortality,n,%	66 (5.2)	15 (7.4)	51 (4.8)	0.12

CABG denotes coronary artery bypass surgery, EMS denotes emergency medical services, VD denotes vessel disease, LAD denotes left anterior descending artery, RCA denotes right coronary artery, LCX denotes left circumflex artery.

* denotes p value < 0.05

For the overall group, the mean age at presentation was 58.4 ± 12.2 years with male preponderance (86%). The overall median D2B time was 60 minutes and mean D2B time was 68 ± 35 minutes. Delay in D2B occurred in 16% of patients. Median D2B time in the delay group was 112 minutes while that of the non-delay group was 56 minutes.

Patients in the delay group were likely to be older and of female gender. There was no ethnic differences in both groups. The former group also had a significantly longer symptom onset to reperfusion time compared to the latter. They were less likely to be smokers and had a higher prevalence of prior MI. For the overall group, the use of emergency medical services (EMS) as mode of presentation was 61%. Patients in the delay group tend to self-present compared to the non-delay group. The majority of STEMI patients (62%) in our study presented after office-hours with a higher proportion (69%) in the delay group. The incidence of posterior MI was significantly higher in the delay group. The rate of triple vessel disease was also significantly higher in the delay group with a trend towards higher proportion of left main disease. Overall, the rate of cardiogenic shock was 20% but there was no significant difference in the rates for both groups.

The inhospital mortality was 5.2% for the overall group. The inhospital mortality was numerically higher in the delay group (7.4% versus 4.8%, p = 0.12) but this was not statistically significant.

Table 2 outlines the reasons for D2B delay for the 202 patients. The most common cause of D2B delay was due to delay in the ED (39%), atypical clinical presentation (37.6%), unstable medical condition requiring stabilization or computed tomographic (CT) imaging, (26.7%), difficulty in crossing culprit lesion (14.3%), difficult vascular access (9.4%), consent issues (3.9%) and “unknown”(9%).

**Table 2:** Reasons for delay in D2B time.

		N (%)
**1**	Delay in emergency department*	79 (39.1)
**2**	Atypical clinical presentation*	76 (37.6)
**3**	Difficult vascular access*	19 (9.4)
**4**	Difficult crossing culprit lesion*	29 (14.4)
**5**	Unstable medical condition requiring stabilization and CT imaging*	54 (26.7)
**6**	Issues with consent*	8 (4)
**7**	Unknown reason	18 (9)

*Patients can have more than 1 reason for D2B delay

To the best of our knowledge, this is the first report on the frequency and causes of delay in PPCI for STEMI patients in a contemporary South-east Asian registry. Delay in D2B occurred in 16% of patients and the median D2B time for the delay group was 112 minutes. From our study, we identified specific clinical characteristics that were associated with D2B delay. Patients in the delay group were older and of female gender compared to the non-delay group. The former group also had a significantly longer symptom onset to reperfusion time. They were less likely to be smokers and had a higher rate of prior MI. This is similar with the findings of other studies [7,9,11] which had shown these age and gender predisposition to longer symptom onset to reperfusion and D2B time. One possible explanation is that the elderly [13–14] and female [15–16] patients tend to have atypical symptom during AMI which could lead to delay in seeking medical consultation, delay in medical attention and missed diagnosis in the ED. In addition, the elderly patients may have impairment in their hearing, visual and cognitive functions which preclude effective communication and proper consent taking. Patients in the delay group were found to have a higher proportion of previous MI, hence, they may have pre-existing ECG changes which could mask their underlying diagnosis of STEMI.

Singapore is an island city-state in South-east Asia which has a population of 5.6 million. According to the government census report, the ethnic composition of Chinese, Malays and Indians in the general population were 74.2%, 13.4% and 9.2% respectively. There was no ethnic differences between the two groups. This suggest that all ethnic groups in Singapore receive equitable access to medical care and is in contrast to other studies [7,11] which had shown that the nonwhite race was a predictor of D2B delay. In our study, patients in the delay group tend to self-present and present after office hours when compared to the non-delay group. Both factors [7–9] have been well reported in the literature to be associated with D2B delay. Possible reasons include delay in triaging, evaluation and diagnosis in the ED. Staffing levels maybe lower after office hours which could contribute to the delay. This might warrant a more detailed analysis of the emergency visit pattern at our institution and redistribution of manpower to better match arrival patterns of patients in order to reduce process-induced delays and improve D2B time.

The incidence of posterior MI was significantly higher in the delay group. This is likely due to difficult interpretation of the subtle ECG changes in posterior MI [17] because no specific leads of the standard ECG directly represent that area. We should highlight this important finding to the emergency physician and educate all relevant staff in order to increase their diagnostic suspicion of posterior MI. Another possible solution is to perform a routine posterior electrocardiogram for individuals with prominent ST-segment depression in the anterior leads (V1 to V3). The rate of triple vessel disease was significantly higher in the delay group with a trend towards higher proportion of left main disease. This suggest the former group had more extensive coronary artery disease.

### D2B and mortality

In our study, the inhospital mortality was 5.2% for the overall group. The inhospital mortality was numerically higher in the delay group but this figure was not statistically significant. This finding is in contrast with previous studies [2–5] that have demonstrated that delay in D2B time are associated with adverse outcomes. A recent study [18], however, have implied a possible threshold limit to D2B time in which further reduction does not impact mortality. The importance of symptom onset to reperfusion [19] ie total ischaemic time may explain why lower D2B time does not always translate to lower inhospital mortality especially if the symptom onset to reperfusion time is already substantial. In our study, the overall mean symptom onset to reperfusion time was 251.3 ± 218 minutes. Those in the non-delay group has a time of 240.3 ± 215 minutes (4 hours before medical contact) which is significantly longer than those reported in the literature [19]. This highlight the importance of public education for seeking immediate medical attention at the onset of chest pain.

### Reasons for delay

We found that the 3 most frequent causes of delay was delay in the ED (39%), atypical clinical presentation (37.6%) followed by unstable medical condition requiring stabilization or CT imaging (26.7%). Delay in the ED [8,10] includes key operational issues like delay in triage, evaluation and diagnosis. Prompt data feedback of time intervals comprising D2B time with staff in ED and catheterization laboratory team is recognized as one of the six strategies [12] for reducing D2B time but it is not practiced at our institution. This should be implemented so that synergistic cooperation between the 2 main stake-holders can be improved and system solutions to the D2B delay can be conceived. For atypical clinical presentation, this includes patients with atypical symptom and nondiagnostic initial ECG. Several studies [8,10] have shown that such features often lead to prolonged D2B. This suggests increased index of suspicion especially in ambiguous cases. Formal training in the assessment of acute coronary syndrome to triage staff members in the ED could be provided regularly to improve their diagnostic acumen. Another potential solution is to perform an ECG for patients presenting with suspicious symptom anywhere from the neck to umbilical area.

For unstable medical condition requiring medical stabilization or CT imaging, these are patients who require intubation due to respiratory distress, cardiac arrest requiring cardiopulmonary resuscitation and those who require CT to rule out aortic dissection or intracranial event. Prior studies [10–11] have shown that these group of patients especially those with cardiac arrest have the highest inhospital mortality. Other contributory causes of D2B delay include technical difficulties in the cardiac catheterization laboratory (delay in vascular access and delay in crossing culprit lesion). Our rates are different from those reported in the literature [11]. However it is difficult to make valid comparisons as patients’ baseline clinical and angiographic characteristics maybe different. 3.9% of patients with D2B delay had issues with giving consent. Based on the documentation in the clinical notes, all the patients wanted further discussion with their family members before agreeing to PPCI. Interestingly, a study conducted in USA [11] using data from Cath-PCI Registry found that most of the patients who had delays in consent were of Asian origin. In the Asian culture, relationships are often family-centric. Decision making in medical care are commonly shared between various members of the family and the patient. Lastly, no specific reason for D2B delay was found in 9% of patients.

### Limitation

Our study had several limitations. In comparison to other studies, our sample size was relatively small. In addition, our study was a retrospective, single-centre observational study, therefore, selection bias was inevitable and could affect our findings. There was a proportion of patients in our study who had delay in D2B time that were classified under “others”. There was no clear documentation of what caused the delay, hence, we were unable to analyze the reasons for delay in D2B time. However, this only constituted a small percentage of our study population. We also did not capture data on the socio-economic status and the prevalence of pre-existing risk factors like prior stroke, chronic renal disease and chronic obstructive pulmonary disease which could affect the outcomes.

### Future direction

Bradley et al [12] identified 6 key strategies for reducing D2B time which include activating the cardiac catheterization laboratory on the basis of ECG performed while patient is on the way to hospital, emergency physician directly activate the catheterization laboratory, having a single call to a central operator to activate the on-call team, staff expected to arrive within 20 minutes after being activated, having on-site cardiologist and prompt data feedback.

The first strategy mentioned above is currently not done as a standard practice at our institution. With the advent of telemedicine and healthcare technology, we should embrace these advances and implement this key strategy in our PPCI program in order to reduce D2B delay and hopefully achieve better clinical outcomes for our STEMI patients.

## Conclusions

Our registry showed that delay in D2B occurred in 16% of our patients undergoing PPCI for STEMI but this did not translate into higher inhospital mortality. Various factors (patient, system and nonsystem-related) for D2B delay unique to the hospital and the South-east Asian patient population were identified and warrant further intervention.

## References

[1] Keeley EC, Hillis LD. Primary PCI for myocardial infarction with ST-segment elevation. N Engl J Med. 2007 1 4;356(1):47–54. doi: 10.1056/NEJMct063503 17202455 10.1056/NEJMct063503

[2] Berger PB, Ellis SG, Holmes DR Jr, Granger CB, Criger DA, Betriu A, et al Relationship between delay in performing direct coronary angioplasty and early clinical outcome in patients with acute myocardial infarction: results from the Global Use of Strategies to Open Occluded Arteries in Acute Coronary Syndromes (GUSTO-IIb) Trial. Circulation 1999; 100:14–20. doi: 10.1161/01.CIR.100.1.14 10393675 10.1161/01.cir.100.1.14

[3] Cannon CP, Gibson CM, Lambrew CT, Shoultz DA, Levy D, French WJ, et al Relationship of symptom-onset-to-balloon time and door-to-balloon time with mortality in patients undergoing angioplasty for acute myocardial infarction. JAMA. 2000 6 14;283(22):2941–7. doi: 10.1001/jama.283.22.2941 10865271 10.1001/jama.283.22.2941

[4] McNamara RL, Wang Y, Herrin J, Curtis JP, Bradley EH, Magid DJ, et al; NRMI Investigators. Effect of door-to-balloon time on mortality in patients with ST-segment elevation myocardial infarction. J Am Coll Cardiol. 2006 6 6;47(11):2180–6. doi: 10.1016/j.jacc.2005.12.072 16750682 10.1016/j.jacc.2005.12.072

[5] Rathore SS, Curtis JP, Chen J, Wang Y, Nallamothu BK, Epstein AJ, et al; National Cardiovascular Data Registry. Association of door-to-balloon time and mortality in patients admitted to hospital with ST elevation myocardial infarction: national cohort study. BMJ. 2009 5 19;338:b1807 doi: 10.1136/bmj.b1807 19454739 10.1136/bmj.b1807 PMC2684578

[6] Levine GN, Bates ER, Blankenship JC, Bailey SR, Bittl JA, Cercek B, et al 2015 ACC/AHA/SCAI Focused Update on Primary Percutaneous Coronary Intervention for Patients With ST-Elevation Myocardial Infarction: An Update of the 2011 ACCF/AHA/SCAI Guideline for Percutaneous Coronary Intervention and the 2013 ACCF/AHA Guideline for the Management of ST-Elevation Myocardial Infarction. J Am Coll Cardiol. 2016 3 15;67(10):1235–50. doi: 10.1016/j.jacc.2015.10.005 26498666 10.1016/j.jacc.2015.10.005

[7] Angeja BG, Gibson CM, Chin R, Frederick PD, Every NR, Ross AM, et al; Participants in the National Registry of Myocardial Infarction 2–3. Predictors of door-to-balloon delay in primary angioplasty. Am J Cardiol. 2002 5 15;89(10):1156–61. doi: 10.1016/S0002-9149(02)02296-8 12008167 10.1016/s0002-9149(02)02296-8

[8] Wu EB, Arora N, Eisenhauer AC, Resnic FS. An analysis of door-to-balloon time in a single center to determine causes of delay and possibilities for improvement. Catheter Cardiovasc Interv. 2008 2 1;71(2):152–7. doi: 10.1002/ccd.21315 17985378 10.1002/ccd.21315

[9] Parikh SV, Jacobi JA, Chu E, Addo TA, Warner JJ, Delaney KA, et al Treatment delay in patients undergoing primary percutaneous coronary intervention for ST-elevation myocardial infarction: a key process analysis of patient and program factors. Am Heart J. 2008 2;155(2):290–7. doi: 10.1016/j.ahj.2007.10.021 18215599 10.1016/j.ahj.2007.10.021

[10] Miedema MD, Newell MC, Duval S, Garberich RF, Handran CB, Larson DM, et al Causes of delay and associated mortality in patients transferred with ST-segment-elevation myocardial infarction. Circulation. 2011 10 11;124(15):1636–44. doi: 10.1161/CIRCULATIONAHA.111.033118 21931079 10.1161/CIRCULATIONAHA.111.033118

[11] Swaminathan RV, Wang TY, Kaltenbach LA, Kim LK, Minutello RM, Bergman G, et al Nonsystem reasons for delay in door-to-balloon time and associated in-hospital mortality: a report from the National Cardiovascular Data Registry. J Am Coll Cardiol. 2013 4 23;61(16):1688–95. doi: 10.1016/j.jacc.2012.11.073 23500264 10.1016/j.jacc.2012.11.073

[12] Bradley EH, Herrin J, Wang Y, Barton BA, Webster TR, Mattera JA, et al Strategies for reducing the door-to-balloon time in acute myocardial infarction. N Engl J Med. 2006 11 30;355(22):2308–20. doi: 10.1056/NEJMsa063117 17101617 10.1056/NEJMsa063117

[13] Gregoratos G. Clinical manifestations of acute myocardial infarction in older patients. Am J Geriatr Cardiol. 2001 Nov-Dec;10(6):345–7. doi: 10.1111/j.1076-7460.2001.00641.x 11684919 10.1111/j.1076-7460.2001.00641.x

[14] Bhatia LC, Naik RH. Clinical profile of acute myocardial infarction in elderly patients. J Cardiovasc Dis Res. 2013 6;4(2):107–11. doi: 10.1016/j.jcdr.2012.07.003 24027366 10.1016/j.jcdr.2012.07.003 PMC3770124

[15] Pope JH, Aufderheide TP, Ruthazer R, Woolard RH, Feldman JA, Beshansky JR, et al Missed Diagnoses of Acute Cardiac Ischemia in the Emergency Department. N Engl J Med. 2000 4 20;342(16):1163–70. doi: 10.1056/NEJM200004203421603 10770981 10.1056/NEJM200004203421603

[16] Ricci B, Cenko E, Varotti E, Puddu PE, Manfrini O. Atypical Chest Pain in ACS: A Trap Especially for Women. Curr Pharm Des. 2016;22(25):3877–84. doi: 10.2174/1381612822666160309115125 26956231 10.2174/1381612822666160309115125

[17] Rich MW, Imburgia M, King TR, Fischer KC, Kovach KL. Electrocardiographic diagnosis of remote posterior wall myocardial infarction using unipolar posterior lead V9. Chest. 1989 9;96(3):489–93. doi: 10.1378/chest.96.3.489 2788559 10.1378/chest.96.3.489

[18] Menees DS, Peterson ED, Wang Y, Curtis JP, Messenger JC, Rumsfeld JS, et al Door-to-balloon time and mortality among patients undergoing primary PCI. N Engl J Med. 2013 9 5;369(10):901–9. doi: 10.1056/NEJMoa1208200 24004117 10.1056/NEJMoa1208200

[19] Bates ER, Jacobs AK. Time to treatment in patients with STEMI. N Engl J Med. 2013 9 5;369(10):889–92. doi: 10.1056/NEJMp1308772 24004114 10.1056/NEJMp1308772

